# Person-centered antenatal care and associated factors in Rwanda: a secondary analysis of program data

**DOI:** 10.1186/s12884-021-03747-z

**Published:** 2021-04-10

**Authors:** Phoebe Miller, Patience A. Afulani, Sabine Musange, Felix Sayingoza, Dilys Walker

**Affiliations:** 1 University of California San Francisco, San Francisco, USA; 2 University of Rwanda School of Public Health, Kigali, Rwanda; 3 Rwanda Biomedical Center, Kigali, Rwanda

**Keywords:** Person-centered, Antenatal care, Maternal health, Person-centered antenatal care

## Abstract

**Background:**

Research suggests that women’s experience of antenatal care is an important component of high-quality antenatal care. Person-centered antenatal care (PCANC) reflects care that is both respectful of, and responsive to, the preferences, needs, and values of pregnant women. Little is known in Rwanda about either the extent to which PCANC is practiced or the factors that might determine its use. This is the first study to quantitatively examine the extent of and the factors associated with PCANC in Rwanda.

**Methods:**

We used quantitative data from a randomized control trial in Rwanda. A total of 2150 surveys were collected and analyzed from 36 health centers across five districts. We excluded women who were less than 16 years old, were referred to higher levels of antenatal care or had incomplete survey responses. Both bivariate and multivariate logistic regression analyses were used to test the hypothesis that certain participant characteristics would predict high PCANC.

**Results:**

PCANC level was found to be sub-optimal with one third of women leaving antenatal care (ANC) with questions or confused and one fourth feeling disrespected. In bivariate analysis, social support, greater parity, being in the traditional care (control group), and being from Burera district significantly predict high PCANC. Additionally, in the multivariate analysis, being in the traditional care group and the district in which women received care were significantly associated with PCANC.

**Conclusions:**

This quantitative analysis indicates sub-optimal levels of PCANC amongst our study population in Rwanda. We find lower levels of PCANC to be regional and defined by the patient characteristics parity and social support. Given the benefits of PCANC, improvements in PCANC through provider training in Rwanda might promote an institutional culture shift towards a more person-centered model of care.

## Background

Despite global progress in reducing maternal mortality, Sub-Saharan Africa accounts for the majority of pregnancy and childbirth related deaths [1]. In 2017, approximately 300,000 women around the world died from pregnancy and childbirth complications; 200,000 of these deaths occurred in Sub-Saharan Africa. Antenatal care (ANC) provides an opportunity for health care providers to positively impact maternal health [2]. ANC ensures early detection, monitoring and treatment of hypertensive disorders of pregnancy, gestational diabetes, and anemia, which may result in the prevention of morbidity and mortality amongst pregnant women [3]. High quality ANC includes not only the provision of these necessary services but also a positive experience of care [4]. According to the World Health Organization (WHO), high quality ANC should be “safe, effective, timely, efficient, equitable and people-centered.” [5].

Globally, women who are young, uneducated and poor are less likely to receive high quality health care [6]. Person-centered care, which “engag [es] women and communities in health care to improve the quality of patient experience and patient-provider interactions” is one important aspect of addressing maternal health inequities [7]. A growing body of literature highlights that abusive or disrespectful maternal care—and not only access to care—may limit improvements in maternal mortality outcomes [8]. Thus, person-centered antenatal care (PCANC) provides one possible strategy within a multifaceted approach for addressing maternal deaths in Sub-Saharan Africa through the delivery of health care which is both respectful of, and responsive to, the preferences, needs, and values of women [9]. PCANC extends the work on person-centered maternity care during childbirth (PCMC) to the pregnancy period, and thus focuses on dignity, respect, support and autonomy with regards to each woman’s health related decision making [10]. The concept and benefits of PCMC are relevant and applicable to PCANC [11, 12]. PCANC has been shown to positively influence the decisions of pregnant women in Sub-Saharan Africa to pursue a facility-based birth and to obtain care from a skilled birth attendant [13–15].

Despite the potential benefits of PCANC and PCMC, disrespect and abuse of women during pregnancy and childbirth are common [16]. In 2015, one study in Kenya and another in Tanzania were the first to demonstrate the high prevalence of abuse and disrespect of pregnant women by health care providers during facility-based births [17, 18]. In Tanzania, the long term impact of abuse during birth was that women were less likely to choose a facility based birth in the future [18]. Since then, most of the research subsequently focused on disrespect and abuse during childbirth [11, 19–23]. Very few studies have examined PCANC, although one study in Kenya demonstrated key gaps in PCANC [9]. Additionally, the majority of studies on quality of ANC focused on the service provision dimensions of quality care [21, 24–28]. In Rwanda, studies have focused generally on provider knowledge and service provision without describing women’s experiences with ANC delivery or the factors associated with PCANC [26, 28].

The primary aim of this paper is to assess the extent of PCANC received by women that participated in a Group Antenatal Care randomized control trial in Rwanda and to examine the factors associated with high and low levels of PCANC. Based on prior studies, we hypothesized that women’s age, socioeconomic status and empowerment would be associated with PCANC [9]. With this analysis, we hope to further inform and revitalize a focus on accountability to the provision of PCANC in Rwanda.

## Methods

### Data collection

**Fig. 1:**
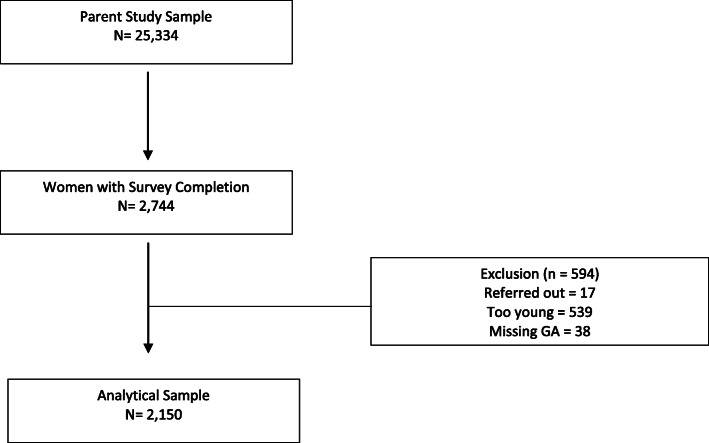
Analytical sample

We conducted a secondary analysis using data from a pair-matched cluster randomized control trial (CRCT) in Rwanda [29]. In the parent study, health centers were randomized to Group Antenatal Care (GANC) or to the standard models of focused antenatal care (ANC) and postnatal care (PNC). A total of 25,334 women were enrolled. As part of the parent study, demographic information was collected for all participants and a convenience sample of the first five women to present to ANC each month were invited to participate in a longer baseline survey. This survey included measurements of satisfaction with care and perceived social support among others. A total of 2744 women completed this baseline survey. For our secondary analysis, we linked demographic information to data collected in the baseline survey. We excluded women who were less than 16 years old, were referred to higher levels of antenatal care or had incomplete survey responses. After exclusion, our analytical sample included a total of 2150 women (Fig. 1).

Baseline surveys were collected in collaboration with the East Africa Preterm Birth Initiative-Rwanda, the Rwanda Ministry of Health, the Rwanda Biomedical Center, the University of Rwanda School of Public Health, and the University of California, San Francisco, Institute of Global Health Sciences. Data collection occurred between May 2017 and December 2018 at 36 different health centers across five districts in Rwanda: Burera, Bugesera, Nyamasheke, Nyarugenge and Rubavu.

We report according to STROBE guidelines [30].

### Variables

Three questions were included in the survey to measure the extent of PCANC delivered to these women based on reported themes of mistreatment during facility-based births and determinants of women’s satisfaction during maternal healthcare [7, 10, 31]: (1) I left the antenatal care visit with questions or confused about some things, (2) The provider did not show me respect or act in respectful ways, and (3) I had enough time with my provider. The complete surveys are published in Musange et al. [32] Initially, responses to these three questions were coded as strongly agree (code = 1), agree (code = 2), neither (code = 3), disagree (code = 4) or strongly disagree (code = 5). Questions were recoded so that code = 1 corresponded with the lowest level of PCANC and code = 5 corresponded with the highest level. We created a summative PCANC score ranging from 3 to 15 by combining responses to the three questions. We then recoded this as a binary variable using the median. Responses 3–11 were coded as low PCANC (code = 0) and 12–15 were coded as having high PCANC (code = 1).

Past research demonstrates that age, socioeconomic background and empowerment are associated with person-centered care during childbirth [19, 33, 34]. We therefore included the following socio-demographic characteristics as has been done in previous research and based on data availability: age, gravidity, parity, education level, health insurance, food security, trimester upon presentation, and, ubudehe category at the first ANC visit. Ubudehe are economic categories designed by the government and designated by communities where category one represents the poorest household and category four the richest [35]. Two forms of empowerment were measured: Whether or not women were able to discuss the health of their pregnancy with their partners was used as a potential measure of social support, and whether or not women brought income to their households was used as a potential measure of financial independence. Having food insecurity was measured as responding yes to any of three questions: have you ever had no food, run out of food, not had enough food.

### Statistical analysis

The analytical sample is 2105. This includes women who had at least one ANC visit during their pregnancy and who completed the survey. Our primary analysis was to examine the extent of PCANC and to determine the factors associated with high PCANC. Frequency distributions were used to describe the background characteristics of the women. Cross-tabulations were used to investigate associations between both the women’s experience with their first ANC visit and factors associated with high PCANC. Pearson’s chi-squared (χ2) tests were used to examine the significant differences between level of PCANC and the explanatory variables. Both bivariate and multivariate logistic regression analyses were used to test the hypothesis that certain participant characteristics would predict high PCANC. Given that data collection happened within the context of a CRCT, within the multivariate analysis, we controlled for the trial group and district. Results are presented in the form of Odds Ratios (OR) reporting 95% confidence intervals. The level of statistical significance using p -values was set at *p* < 0.05. All analyses were performed using Stata/SE for Mac (updated March 2018).

### Ethical considerations

This secondary analysis was approved as exempt and used data solely collected in the parent study. The parent study protocol was reviewed and approved by the UCSF Institutional Review Board (#16–21,177, 2017) and the Rwanda National Ethics Committee (No 0034/RNEC/2017). A waiver of parental consent for any adolescent 15 years of age or older was granted by the Rwanda National Ethics Committee, allowing adolescents over 15 to consent to participation in primary and secondary interventions and data collection and analysis.

Data collectors obtained written, or if illiterate, verbal consent from participants prior to conducting the oral survey. Participants were given a printed consent form to read while it was also read to them in Kinyarawanda (local language), assuring those participants who were illiterate were understanding the content. The consent statement explained the study objectives, requirements, potential risks, privacy and ethical obligations of the research team. Participants participated in the study if they agreed to sign the consent statement.

All members of the research team were trained in ethical practices in human research. Research staff emphasized that participation in the study was voluntary and that refusal to participate in the study would not result in negative repercussions. This study was registered on ClinicalTrials.gov, ID NCT03154177 on May 16, 2017. All data from the parent study was de-identified.

## Results

Table 1 presents the sociodemographic characteristic of the sample. The average age of women was 28 years. One fourth of the women were primigravida (27%). The majority (75%) had only a primary education or less. A minority (8%) of women reported having no health insurance and most (63%) of those insured had *Mutuelle* (a form of community-based health insurance). The majority of women sampled were unable to contribute financially to their households (77%) but most were able to discuss the health of their pregnancy with their partners (88%). The majority reported food insecurity (62%). The sample was distributed between the five districts (Burera, Bugesera, Nyamasheke, Nyarugenge and Rubavu) with the majority of participants in Bugesera (31%) and the minority in Nyarugenge (11%).

**Table 1:** Demographics and reproductive health characteristics of study participants, overall (*N* = 2150)

Characteristics	N (%)
Age	
16–19	60 (2.8)
20–24	581 (27.0)
25–29	584 (27.2)
30–34	523 (24.3)
35+	402 (18.7)
Gravidity	
Primigravida	564 (26.2)
Multigravida	1581 (73.5)
No Response	5 (0.2)
Parity	
Nulliparous	611 (28.4)
Multiparous	1581 (73.5)
No Response	4 (0.2)
Trimester	
First	413 (20.6)
Second	1588 (79.4)
Third	149 (6.9)
Education	
None	160 (7.4)
Primary or less	1461 (67.9)
Secondary or more	515 (23.9)
No Response	14 (0.6)
Health Insurance^a^	
No Insurance	175 (8.1)
CBHI	1350 (62.8)
RSSB	46 (2.1)
MMI	15 (0.7)
Other	1 (0.05)
No Response	563 (26.2)
Ubudehe Category	
1 (lowest)	308 (14.3)
2	606 (28.2)
3	544 (25.3)
4 (highest)	0 (0.0)
Unknown	692 (28.0)
Discusses Pregnancy with Partner	
Yes	1902 (88.5)
No	221 (10.3)
No Response	27 (1.3)
Contributor to Household Finances	
Yes	437 (20.3)
No	1661 (77.3)
No Response	50 (2.5)
Food Secure	
Yes	1343 (62.5)
No	807 (37.5)
No Response	0 (0.0)
Study Group	
Group Care	976 (45.4)
Traditional Care	1174 (54.6)
HC District	
Burera	364 (16.9)
Bugesera	664 (30.9)
Nyamasheke	518 (24.1)
Nyarugenge	230 (10.7)
Rubavu	365 (17.0)
No Response	9 (0.4)

Notes

All percentages are column percentage

^a^ CBHI is *Mutuelle* (Community-Based Health Insurance); RSSB is the Rwanda Social Security Board; MMI is military medical insurance

Table 2 presents the frequency of PCANC. Thirty-percent of women left the ANC visit with questions or confused about some things, 23% reported disrespect from their provider, and 3% reported not having enough time with their provider. The average PCANC score based on the three questions was 11.33 (SD: 2.44, Range: 5–15, Median: 12). Forty-five percent of responses scored between 3 and 11, which were recoded to low PCANC, the remaining (score of 12–15) were categorized as high PCANC.

**Table 2:** Person-Centered Antenatal Care (PCANC) Frequency

PCANC Measure	High PCANC	Low PCANC
	N(%)	N(%)
Experienced Respect	1639 (76.3)	509 (23.7)
Left with Questions	1493 (69.4)	657 (30.6)
Not Enough Time	2082 (96.9)	67 (3.1)
Combined Score	1185 (55.1)	965 (44.9)

Table 3 presents the results of bivariate associations with chi-square tests for the relationship between PCANC at the first ANC visit and selected factors. Factors that were significantly associated with high PCANC in the bivariate analysis were multiparity and being able to discuss the health of one’s pregnancy with one’s partner. The factors that were associated with low PCANC were study group and receiving care in any district other than Burera. Women who were multiparous (56%) were more likely to have high PCANC than were those who were nulliparous (51%). Women who were able to discuss the health of their pregnancies with their partners were more likely to have high PCANC (56%) than were women who did not discuss pregnancies with partners (47%). The districts where women were most likely to receive high PCANC were Burera (72%) and Rubavu (59%) compared to 45% in Nyamasheke district which had the lowest PCANC. Those who were allocated to receive exclusively traditional care after their first visit were more likely to receive high PCANC (69%) during their first visit than were those who would receive exclusively group care after their first visit (39%).

**Table 3:** Person-Centered Antenatal Care (PCANC) according to sociodemographic and reproductive health characteristics

Characteristics	High PCANC^a^	*P* Value^b^
	N(%)	
Total	1185 (55.1)	
Age		0.546
16–19	32 (53.3)	
20–24	321 (55.2)	
25–29	306 (52.4)	
30–34	295 (56.4)	
35+	231 (57.5)	
Gravidity		0.083
Primigravida	294 (52.1)	
Multigravida	891 (56.4)	
Parity		0.020
Nulliparous	313 (51.2)	
Multiparous	871 (56.7)	
Trimester		0.046
First	242 (58.6)	
Second	851 (53.6)	
Third	92 (61.7)	
Education		0.749
None	92 (57.5)	
Primary or less	809 (55.4)	
Secondary or more	279 (54.2)	
Health Insurance^c^		0.486
No Insurance	104 (59.4)	
CBHI	820 (60.7)	
RSSB	33 (71.7)	
MMI	8 (53.3)	
Other	1 (100.0)	
Ubudehe Category		0.376
1 (Lowest)	187 (60.7)	
2	358 (59.1)	
3	343 (63.0)	
4 (Highest)	0 (0.0)	
Unknown	49 (55.1)	
Discusses Pregnancy with Partner		0.013
Yes	1070 (56.3)	
No	105 (47.5)	
Contributor to Household Finances		0.519
Yes	248 (56.7)	
No	914 (55.0)	
Food Secure		0.985
Yes	740 (55.1)	
No	445 (55.1)	
Study Group		0.000
Group Care	377 (38.6)	
Traditional Care	808 (68.8)	
HC District		0.000
Burera	261 (71.7)	
Bugesera	358 (53.9)	
Nyamasheke	232 (44.8)	
Nyarugenge	116 (50.4)	
Rubavu	216 (59.2)	

Note:

All percentages are row percentages

^a^ High PCANC defined as summative scores between 11 and 15

^b^ *P* Values are from bivariable Pearson chi-square tests

^c^ CBHI is *Mutuelle* (Community-Based Health Insurance); RSSB is the Rwanda Social Security Board; MMI is military medical insurance

Table 4 shows results from both bivariate and multivariate regression models. In the bivariate model multiparity was significantly correlated with having high PCANC (OR 1.24 [1.03–1.51]). Ability to discuss one’s pregnancy with one’s partner was also correlated with having high PCANC in the bivariate model (OR 1.42 [1.07–1.88]). Receiving care at a facility trained for group care was significantly associated with low PCANC in the bivariate model (OR 0.28 [0.24–0.33]). In the multivariate regression, receiving care in Bugesera (OR 0.55 [0.39–0.78]), Nyamasheke (OR 0.411 [0.28–0.59]), Nyarugenge (OR 0.55 [0.36–0.85]) or Rubavu (OR 0.47 [0.32–0.70]) were associated with low PCANC when compared to Burera. Being assigned to group care also remained significantly associated with low PCANC (OR 0.32 [0.25–0.40]) in the multivariate regression.

**Table 4:** Predictors of High Levels of Person-Centered Antenatal Care (PCANC)

Characteristics	Bivariate PCANC	Multivariate PCANC
	OR [95% CI]	OR [95% CI]
Age		
16–19	Ref.	Ref.
20–24	1.08 [0.63–1.84]	1.34 [0.71–2.52]
25–29	0.96 [0.56–1.64]	1.13 [0.59–2.19]
30–34	1.13 [0.66–1.93]	1.47 [0.74–2.91]
35+	1.18 [0.68–2.03]	1.32 [0.66–2.67]
Parity		
Nulliparous	Ref.	Ref.
Multiparous	1.24 [1.03–1.51] **	1.23 [0.90–1.68]
Gestational Age		
First	Ref.	Ref.
Second	0.81 [0.65–1.01]*	0.82 [0.63–1.67]
Third	1.14 [0.78–1.67]	0.82 [0.50–1.32]
Education Level		
None	Ref.	Ref.
Primary	0.92 [0.66–1.27]	1.15 [0.75–1.76]
Secondary or more	0.87 [0.61–1.25]	1.37 [0.86–2.24]
Ubudehe Category		
1 (Lowest)	Ref.	Ref.
2	0.93 [0.70–1.24]	0.93 [0.68–1.26]
3	1.10 [0.83–1.47]	0.99 [0.72–1.36]
4 (Highest)	–	–
Unknown	0.79 [0.49–1.27]	0.84 [0.51–1.41]
Partner discussion		
No	Ref.	Ref.
Yes	1.42 [1.07–1.88]**	1.12 [0.76–1.66]
Contributes to household finances		
No	Ref.	Ref.
Yes	1.08 [0.87–1.33]	1.23 [0.85–1.77]
Study Group		
Traditional Care	Ref.	Ref.
Group Care	0.28 [0.24–0.34]**	0.32 [0.25–0.40]**
HC District		
Burera	Ref.	Ref.
Bugesera	0.46 [0.35–0.61]**	0.55 [0.39–0.78]**
Nyamasheke	0.32 [0.24–0.43]**	0.411 [0.28–0.59]**
Nyarugenge	0.40 [0.28–0.57]**	0.55 [0.36–0.85]**
Rubavu	0.57 [0.42–0.78]**	0.47 [0.32–0.70]**

Notes

**p < 0.05

*p < 0.1

## Discussion

The primary objective of this secondary analysis is to assess the extent of PCANC received by women who participated in a randomized control trial in Rwanda and to explore factors associated with receiving PCANC. To our knowledge this is the first study in Rwanda to quantitatively examine factors associated with PCANC. We find that PCANC is sub-optimal with 30% of women leaving ANC either with questions or confused and 24% feeling disrespected. In bivariate analysis, factors that significantly predict high PCANC are better levels of social support, greater parity, being in the traditional care (control group), and being from Burera district. However, only the study group to which women were assigned and district in which women received care were significantly associated with low PCANC in the multivariate analysis. Our findings are consistent with prior studies on quality of ANC and person-centered care during childbirth, but differ in some ways.

That 30% of women left ANC with questions or confused adds to the evidence on critical gaps in communication during ANC and childbirth [9, 12, 36]. In a study in Kenya on PCANC, about one-third of women did not often understand the purposes of tests and medicines received and did not feel able to ask questions to the health care provider [9]. Similarly, in a study in Nigeria on ANC, more than half of women could not recall any danger signs of pregnancy that would compel them to return to the doctor and more than a quarter reported wait times to see a provider of greater than 2 h [36]. The rate of disrespect during antenatal care found in our study is also similar to rates of self-reported disrespect during facility-based childbirth in Rwanda [22, 37]. A cross-sectional household study in Rwanda found that 22.5% of women felt disrespected during childbirth [22, 37]. The low levels of person-centered care during pregnancy in our study as well as during childbirth reported in other studies in Rwanda might reflect multiple causes such as hierarchical authority structures that legitimize coercive and rushed attitudes, staffing and resource constraints, and a lack of oversight, which are the same for both ANC and birthing facilities [19, 38]. Additionally, our findings may be overestimating the extent of high PCANC, given evidence that women tend to underreport disrespect and abuse because they have not been exposed to medical systems that are sensitive to their humanity and may normalize disrespectful care [39]. Similarly, in a Nigerian study women reported greater than 90% satisfaction with care despite evidence of long wait times and poor patient education [36]. Disrespect may also be invisible due to long standing patterns of poor quality care in the context of resource scarcity [16, 40].

Our finding in the bivariate analysis that being socially supported—the ability to discuss the health of one’s pregnancies with one’s partner—was associated with high PCANC is also consistent with prior studies. Multiple studies demonstrate that empowerment broadly promotes the use of recommended health services [25, 41] and social support, more specifically, has a protective effect [17]. Studies on person-centered care during childbirth also report high person-centered care among more empowered women [20, 21]. Notably, however, these studies also find financial autonomy to be a significant factor, which was not significant in our analysis. This might be because of the nearly universal health insurance scheme in Rwanda or because our sample was more representative of economically disadvantaged women [41, 42], which will make a woman’s ability to advocate for herself a more important determinant of the extent of PCANC she receives when compared to others in our study. This highlights the potential benefit of a greater understanding of the complexity of empowerment measures and of evaluating multiple axes of empowerment [41] when describing quality of care.

Additionally, we found that greater parity contributed to higher quality of care in our bivariate model. This differed from other studies in Nepal and Kenya on service provision, which demonstrate that greater parity results in low quality of care [17, 25]. In both of these studies, the authors suggest that women who had already had successful births experienced complacency around receiving all the necessary services for their current pregnancies. However, in our study, quality is focused exclusively on the experience of care and not service provision, as was the focus of these studies, and, by comparison, qualitative studies on women’s experiences during childbirth support our finding that first time mothers tend to experience more disrespect and abuse [19].

Notably, women in Burera and Rubavu received high PCANC when compared to other districts. Some of the variability might reflect ongoing development projects by partners as well as unaccounted variations in the districts. Additionally, there should not have been a significant difference between those who would receive group care and those who would receive traditional care, given that the first visit was individual, standardized, and did not follow the group care format. The differential effect may be a reflection of the increased time taken for scheduling a woman’s subsequent group care visits in the group care sites, decreasing the time available to focus on the woman’s needs. The additional information to orient them to when, where, and how their subsequent visits would be conducted in the context of limited personnel, which might have resulted in a less person-centered encounter [29] and less time available to illicit questions or concerns from women. There are other factors that may also be playing a role including baseline differences at facilities we were unable to account for. For example, women allocated to the group care study arm might have had high expectations of their care after being oriented to the group care model. As there was no one PCANC question that drove this result, our findings may suggest that the introduction of group care, a complete reconfiguration of how ANC is provided, has the potential to disrupt the provision of core care components, specifically those related to person-centeredness [27]. Further studies are required to better understand this finding.

### Limitations

The study is a secondary analysis of survey data collected within a CRCT designed to evaluate group antenatal care and preterm birth in Rwandan facilities with a model of group care that began after the first individual visit at all facilities. As such, the study was not designed specifically to measure PCANC. We were limited by the available variables relevant to PCANC. For example, other aspects of PCANC such as privacy, confidentiality, autonomy, social support, timeliness, cleanliness [10], or the availability of specific amenities [30] could not be examined because data were not available. Additionally, responses were self-reported, which may have resulted in under-reporting of disrespect [16, 43]. In the future, perhaps visits could be observed or the self-report survey can ask about more specific elements of respect (Did you feel the doctors, nurses, or other health providers shouted at you, scolded, insulted, threatened, or talked to you rudely?) or of the facility upkeep (were there clean sheets, drinking water, electricity etc. …) [17].

## Conclusions

Overall, based on limited measures, we find room for improvement in PCANC amongst our study population in Rwanda. Further exploration of both regional differences as well as provider and patient perceptions of limitations to providing and receiving person-centered care might provide avenues for intervention. Improvements in PCANC might positively impact the perception of care and also improve outcomes. In order to better understand all dimensions of quality ANC—including measures not assessed in this study such as patient privacy and supplies—better measurement tools are needed to more comprehensively measure PCANC. The tools that have been validated for person-centered care during childbirth [10] could be adapted for ANC [9]. We also recommend wider use of these validated tools in national surveys to get a systematic understanding of PCANC in Rwanda and globally. Additionally, we recommend thoughtful measures of empowerment during antenatal care to capture the social experiences of women in Rwanda.

## Data Availability

The datasets used and/or analyzed during the current study are available from the corresponding author on reasonable request.

## Abbreviations

ANC: Antenatal Care
CRCT: Cluster Randomized Control Trial
PCC: Person- Centered Care
PCMC: Person-Centered Maternity Care
PCANC: Person-Centered Antenatal Care

## References

[1] Trends in maternal mortality 2000 to 2017: estimates by WHO, UNICEF, UNFPA, World Bank Group and the United Nations Population Division. Geneva: World Health Organization; 2019.

[2] Carroli G, Rooney C, Villar J (2001). How effective is antenatal care in preventing maternal mortality and serious morbidity? An overview of the evidence. Paediatr Perinat Epidemiol.

[3] Bauserman M, Lokangaka A, Thorsten V, Tshefu A, Goudar SS, Esamai F, Garces A, Saleem S, Pasha O, Patel A, Manasyan A (2015). Risk factors for maternal death and trends in maternal mortality in low-and middle-income countries: a prospective longitudinal cohort analysis. Reprod Health.

[4] Van den Broek NR, Graham WJ (2009). Quality of care for maternal and newborn health: the neglected agenda. BJOG Int J Obstet Gynaecol.

[5] World Health Organization. WHO recommendations on antenatal care for a positive pregnancy experience. Geneva: World Health Organization; 2016. 28079998

[6] Say L, Raine R (2007). A systematic review of inequalities in the use of maternal health care in developing countries: examining the scale of the problem and the importance of context. Bull World Health Organ.

[7] Sudhinaraset M, Afulani P, Diamond-Smith N, Bhattacharyya S, Donnay F, Montagu D. Advancing a conceptual model to improve maternal health quality: the person-centered care framework for reproductive health equity. Gates Open Research. 2017;1. 10.12688/gatesopenres.12756.1. 10.12688/gatesopenres.12756.1 PMC5764229 29355215

[8] Bhutta ZA, Salam RA, Lassi ZS, Austin A, Langer A (2014). Approaches to improve quality of care (QoC) for women and newborns: conclusions, evidence gaps and research priorities. Reprod Health.

[9] Afulani PA, Buback L, Essandoh F, Kinyua J, Kirumbi L, Cohen CR (2019). Quality of antenatal care and associated factors in a rural county in Kenya: an assessment of service provision and experience dimensions. BMC Health Serv Res.

[10] Afulani PA, Diamond-Smith N, Golub G, Sudhinaraset M (2017). Development of a tool to measure person-centered maternity care in developing settings: validation in a rural and urban Kenyan population. Reprod Health.

[11] Downe S, Lawrie TA, Finlayson K, Oladapo OT (2018). Effectiveness of respectful care policies for women using routine intrapartum services: a systematic review. Reprod Health.

[12] Afulani PA, Moyer CA (2019). Accountability for respectful maternity care. Lancet.

[13] Chukwuma A, Wosu AC, Mbachu C, Weze K (2017). Quality of antenatal care predicts retention in skilled birth attendance: a multilevel analysis of 28 African countries. BMC Pregnancy Childbirth..

[14] Adjiwanou V, LeGrand T (2013). Does antenatal care matter in the use of skilled birth attendance in rural Africa: a multi-country analysis. Soc Sci Med.

[15] Afulani PA, Moyer C (2016). Explaining disparities in use of skilled birth attendants in developing countries: a conceptual framework. PLoS One.

[16] Bowser D, Hill K (2010). Exploring evidence for disrespect and abuse in facility-based childbirth.

[17] Abuya T, Warren CE, Miller N, Njuki R, Ndwiga C, Maranga A, Mbehero F, Njeru A, Bellows B (2015). Exploring the prevalence of disrespect and abuse during childbirth in Kenya. PLoS One.

[18] Kujawski S, Mbaruku G, Freedman LP, Ramsey K, Moyo W, Kruk ME (2015). Association between disrespect and abuse during childbirth and women’s confidence in health facilities in Tanzania. Matern Child Health J.

[19] Bohren MA, Vogel JP, Hunter EC, Lutsiv O, Makh SK, Souza JP, Aguiar C, Coneglian FS, Diniz AL, Tunçalp Ö, Javadi D (2015). The mistreatment of women during childbirth in health facilities globally: a mixed-methods systematic review. PLoS Med.

[20] Miller S, Abalos E, Chamillard M, Ciapponi A, Colaci D, Comandé D, Diaz V, Geller S, Hanson C, Langer A, Manuelli V (2016). Beyond too little, too late and too much, too soon: a pathway towards evidence-based, respectful maternity care worldwide. Lancet.

[21] Freedman LP, Ramsey K, Abuya T, Bellows B, Ndwiga C, Warren CE, Kujawski S, Moyo W, Kruk ME, Mbaruku G (2014). Defining disrespect and abuse of women in childbirth: a research, policy and rights agenda. Bull World Health Organ.

[22] Rosen HE, Lynam PF, Carr C, Reis V, Ricca J, Bazant ES, Bartlett LA (2015). Direct observation of respectful maternity care in five countries: a cross-sectional study of health facilities in east and southern Africa. BMC Pregnancy Childbirth..

[23] Solnes Miltenburg A, van Pelt S, Meguid T, Sundby J (2018). Disrespect and abuse in maternity care: individual consequences of structural violence. Reproductive Health Matters.

[24] Afulani PA (2015). Rural/urban and socioeconomic differentials in quality of antenatal care in Ghana. PLoS One.

[25] Joshi C, Torvaldsen S, Hodgson R, Hayen A (2014). Factors associated with the use and quality of antenatal care in Nepal: a population-based study using the demographic and health survey data. BMC Pregnancy Childbirth.

[26] Manzi A, Nyirazinyoye L, Ntaganira J, Magge H, Bigirimana E, Mukanzabikeshimana L, Hirschhorn LR, Hedt-Gauthier B (2018). Beyond coverage: improving the quality of antenatal care delivery through integrated mentorship and quality improvement at health centers in rural Rwanda. BMC Health Serv Res.

[27] Sharma J, Leslie HH, Kundu F, Kruk ME (2017). Poor quality for poor women? Inequities in the quality of antenatal and delivery care in Kenya. PLoS One.

[28] Rurangirwa AA, Mogren I, Ntaganira J, Govender K, Krantz G (2018). Quality of antenatal care services in Rwanda: assessing practices of health care providers. BMC Health Serv Res.

[29] Sayinzoga F, Lundeen T, Gakwerere M, Manzi E, Nsaba YD, Umuziga MP, Kalisa IR, Musange SF, Walker D (2018). Use of a facilitated group process to design and implement a group antenatal and postnatal care program in Rwanda. J Midwifery Women’s Health.

[30] Gallo V, Egger M, McCormack V, Farmer PB, Ioannidis JP, Kirsch-Volders M, Matullo G, Phillips DH, Schoket B, Stromberg U, Vermeulen R (2012). Strengthening the reporting of observational studies in epidemiology–molecular epidemiology (STROBE-ME): an extension of the STROBE statement. Mutagenesis..

[31] Sheferaw ED, Mengesha TZ, Wase SB (2016). Development of a tool to measure women’s perception of respectful maternity care in public health facilities. BMC Pregnancy Childbirth..

[32] Musange SF, Butrick E, Lundeen T, Santos N, Firdaus HA, Benitez A, Nzeyimana D, Murindahabi NK, Nyiraneza L, Sayinzoga F, Ndahindwa V (2019). Group antenatal care versus standard antenatal care and effect on mean gestational age at birth in Rwanda: protocol for a cluster randomized controlled trial. Gates Open Research..

[33] Afulani PA, Sayi TS, Montagu D (2018). Predictors of person-centered maternity care: the role of socioeconomic status, empowerment, and facility type. BMC Health Serv Res.

[34] Afulani PA, Phillips B, Aborigo RA, Moyer CA (2019). Person-centred maternity care in low-income and middle-income countries: analysis of data from Kenya, Ghana, and India. Lancet Glob Health.

[35] Nizeyimana P, Lee KW, Sim S. A study on the classfication of households in Rwanda based on factor scores. 한국데이터정보과학회지 2018;29(2):547–555.

[36] Salomon A, Ishaku S, Kirk KR, Warren CE (2019). Detecting and managing hypertensive disorders in pregnancy: a cross-sectional analysis of the quality of antenatal care in Nigeria. BMC Health Serv Res.

[37] Mukamurigo J, Dencker A, Ntaganira J, Berg M (2017). The meaning of a poor childbirth experience–a qualitative phenomenological study with women in Rwanda. PLoS One.

[38] Mukamurigo JU, Berg M, Ntaganira J, Nyirazinyoye L, Dencker A (2017). Associations between perceptions of care and women’s childbirth experience: a population-based cross-sectional study in Rwanda. BMC Pregnancy Childbirth..

[39] Ishola F, Owolabi O, Filippi V (2017). Disrespect and abuse of women during childbirth in Nigeria: a systematic review. PLoS One.

[40] Kruk ME, Kujawski S, Mbaruku G, Ramsey K, Moyo W, Freedman LP (2018). Disrespectful and abusive treatment during facility delivery in Tanzania: a facility and community survey. Health Policy Plan.

[41] Kabeer N (1999). Resources, agency, achievements: reflections on the measurement of women's empowerment. Dev Chang.

[42] Tran TK, Nguyen CT, Nguyen HD, Eriksson B, Bondjers G, Gottvall K, Ascher H, Petzold M (2011). Urban-rural disparities in antenatal care utilization: a study of two cohorts of pregnant women in Vietnam. BMC Health Serv Res.

[43] Saksena P, Antunes AF, Xu K, Musango L, Carrin G. Impact of mutual health insurance on access to health care and financial risk protection in Rwanda. Geneva: World Health Report; 2010. 10.1016/j.healthpol.2010.09.009 20965602

